# The Impact of Electroconvulsive Therapy on Apoptosis-Related Biomarker Gene Expression in Treatment-Resistant Depression

**DOI:** 10.3390/genes17010057

**Published:** 2026-01-04

**Authors:** Ermin Fetahovic, Dragica Selakovic, Marina Mitrovic, Nemanja Jovicic, Bojana Simovic Markovic, Jovan Milosavljevic, Branimir Radmanovic, Dragan Milovanovic, Biljana Ljujic, Gvozden Rosic, Vladimir Janjic

**Affiliations:** 1 Department of Psychiatry, Faculty of Medical Sciences, University of Kragujevac, 34000 Kragujevac, Serbia; erminfetahovic96@gmail.com (E.F.); biokg2005@yahoo.com (B.R.); vladadok@yahoo.com (V.J.); 2 Department of Communication Skills, Ethics, and Psychology, Faculty of Medical Sciences, University of Kragujevac, 34000 Kragujevac, Serbia; 3 Psychiatric Clinic, University Clinical Center Kragujevac, 34000 Kragujevac, Serbia; 4 Department of Physiology, Faculty of Medical Sciences, University of Kragujevac, 34000 Kragujevac, Serbia; dragica984@gmail.com (D.S.); jovan.milosavljevic1997@gmail.com (J.M.); 5 Department of Medical Biochemistry, Faculty of Medical Sciences, University of Kragujevac, 34000 Kragujevac, Serbia; mitrovicmarina34@gmail.com (M.M.); bojana.simovic@gmail.com (B.S.M.); 6 Center for Molecular Medicine and Stem Cell Research, Faculty of Medical Sciences, University of Kragujevac, 34000 Kragujevac, Serbia; 7 Department of Histology and Embryology, Faculty of Medical Sciences, University of Kragujevac, 34000 Kragujevac, Serbia; nemanjajovicic.kg@gmail.com; 8 Department of Pharmacology and Toxicology, Faculty of Medical Sciences, University of Kragujevac, 34000 Kragujevac, Serbia; piki@fmn.kg.ac.rs; 9 Department of Genetics, Faculty of Medical Sciences, University of Kragujevac, 34000 Kragujevac, Serbia; bljujic74@gmail.com

**Keywords:** major depressive disorder, treatment-resistant depression, electroconvulsive therapy, apoptosis, relative gene expression

## Abstract

**Background/Objectives**: The aim of this study was to simultaneously evaluate alterations in apoptosis-related biomarker gene expression accompanied by electroconvulsive therapy (ECT) in treatment-resistant depression (TRD) patients. **Methods**: A total of 25 subjects (15 healthy controls; 10 TRD patients) were initially tested for baseline values of relative mRNA expression of apoptosis-related markers (Bax, Bcl-2, p53, and cytochrome c) in peripheral blood samples and MADRS score. **Results**: Healthy subjects showed significantly lower values in MADRS, and Bax and p53, with increased Bcl-2 expression. The four-week ECT protocol (bitemporal, three sessions per week, with MADRS evaluation and blood sampling after each week) in TRD patients resulted in a concomitant significant decrease in MADRS, Bax, and p53 and an increase in Bcl-2 expression. **Conclusions**: Our results confirmed that the benefits observed by clinical outcome may also be attributed to the anti-apoptotic impact of ECT.

## 1. Introduction

Major depressive disorder (MDD) is a complex psychiatric condition characterized by persistently low or depressed mood, anhedonia or decreased interest in pleasurable activities, feelings of guilt or worthlessness, lack of energy, poor concentration, appetite changes, psychomotor retardation or agitation, sleep disturbances, or suicidal thoughts [1]. It represents one of the leading causes of disability worldwide, and it is ranked as the second leading cause of years spent with disability, increasing not only the risk of self-harm and suicide but also entailing an increased burden of chronic diseases, including cancer, diabetes, and cardiovascular disease [2]. Globally, it is estimated that approximately 290 million individuals are affected by depression, with higher prevalence among older adults and women. MDD is expected to become the leading contributor to the global burden of disease by 2030 [3].

**Figure 1:**
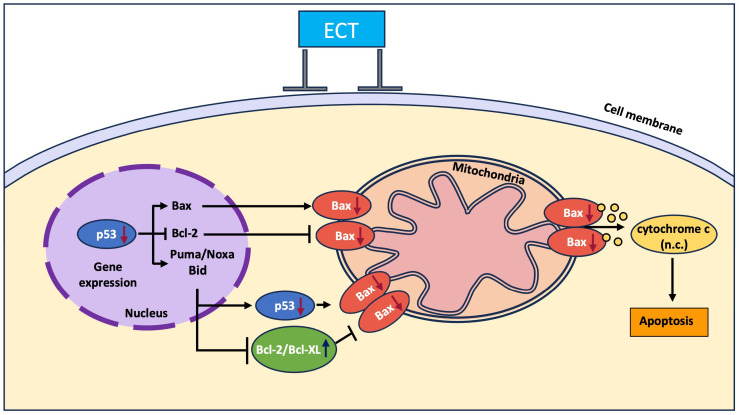
Key apoptotic pathways occurring along with ECT protocol (Bax—BCL2-associated X protein; Bcl-2—B-cell lymphoma 2; Puma/Noxa Bid—p53-Upregulated Modulator of Apoptosis/Phorbol-12-Myristate-13-Acetate-Induced Protein 1; BH3 Interacting-Domain Death Agonist; ↑, ↓, and n.c. represent increase, decrease, and no significant change, respectively).

During the long-lasting period of the MDD pathophysiological background, numerous factors have been identified as causally important. Most proposed mechanisms were classified into several broad hypothesis categories [4], such as hypothalamic–pituitary–adrenal axis dysfunction, the monoamine hypothesis, neuroinflammation, structural and functional brain remodeling, social–psychological factors, and genetic and epigenetic anomalies. Interestingly, several of these theories end with the conclusion that the final mechanism involved in the etiology of MDD includes apoptotic events (Figure 1). Previous clinical trials confirmed the crosstalk between several apoptotic markers (Bcl-2, Bax, and Fas), with a typical algorithm for increased pro-apoptotic (and/or decreased anti-apoptotic) activity that correlates to the severity of depressive disorder [5], as well as to the outcomes of treatment with antidepressants [6]. Even more, there is evidence that the severity of clinical manifestations, including a high risk for suicide, can correlate with the extent of inflammatory–apoptotic acceleration [7].

Treatment of depression requires a comprehensive assessment, as well as the establishment of an appropriate diagnosis. Therapeutic options for the treatment of MDD can be broadly divided into pharmacological and non-pharmacological [8].

Pharmacological strategies are still the first line of treatment for mild to severe MDD, but the percentage of response to antidepressants is moderate (40–60%), and the percentage of remission is even rarer (30–35%), while more than a third of patients do not have a satisfactory response to pharmacotherapy [9]. Once a patient fails to respond to two adequate, different antidepressant trials, the illness is labeled as treatment-resistant depression (TRD). It is currently estimated that at least 30% of people with depression meet this definition [10]. Various non-pharmacological methods exist as treatment options for TRD, such as electroconvulsive therapy, repetitive transcranial magnetic stimulation, magnetic seizure therapy, deep brain stimulation, etc., all showing different efficacy, speed, and duration of action, as well as side effects [11]. Additionally, there is an evident lack of standardization in those therapeutic approaches, which hinders the reliable comparison of their efficiency.

Electroconvulsive therapy (ECT), which dates back almost a century [12], represents the oldest brain stimulation procedure in psychiatry and is associated with rapid response and remission in the majority of patients with resistant and severe depression [13]. Current ECT protocols have shown significant results in reducing suicidal risk, improving patients’ quality of life, functional outcomes, and reducing the number of rehospitalizations [14]. Studies on TRD patients have shown response rates of 60–80% and remission rates of 50–60% in patients who underwent ECT [15,16]. Although the use of this procedure has been on the rise in recent years, studies show that it is still underutilized [17], which may be, in part, the consequence of inadequate evaluation of neurochemical alterations that appear along with ECT.

The molecular mechanisms of MDD and its most effective treatments, including ECT, still remain unclear. Various molecular pathways have been implicated in the effects of ECT. Namely, it has been reported that the benefits of ECT can be attributed to the neurotransmitter modulation, neurotrophic signaling, immune regulation, and diminishing of oxidative stress [18]. Again, many of those molecular mechanisms are accompanied by alterations in apoptotic profile. There is an obvious inconsistency considering the ECT impact on the apoptotic events. Probably due to heterogeneity of the subjects (different species, age, pretreatments, comorbidity, other therapeutic protocols, etc.) and experimental design and protocols (the number of ECT sessions, protocol duration, etc.), there is no general consensus for the ECT-induced apoptotic marker alterations. While Zarubenko and coworkers [19] reported decreased apoptotic indicator levels in the mice hippocampus (but not in the cerebral cortex and cerebellum) after repeated electric shocks, Ito and colleagues [20] observed no anti-apoptotic activity in the rat hippocampus after a prolonged protocol of electroconvulsive stimulation (ECS), despite the initial anti-apoptotic effect of ECS (in a single trial). On the other hand, clinical trials showed no significant alterations in neuronal injury and apoptotic markers [21], although E2F1 mRNA levels (cell survival and proliferation) are significantly lower in the peripheral blood of MDD patients [22].

The aim of this study was to assess alterations in apoptosis-related biomarker gene expression profiles following ECT in TRD and to explore whether ECT modulates the potential relationship between molecular pathways involved in cell survival and clinical outcome.

## 2. Materials and Methods

**Figure 2:**
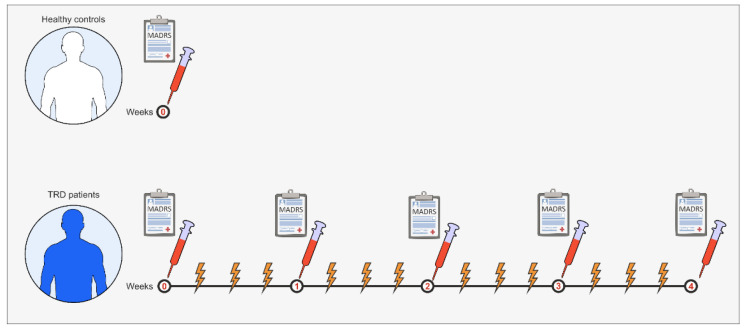
Experimental protocol design.

This study consisted of two groups of subjects: a control group of healthy volunteers (*n* = 15) who underwent blood sampling and clinical evaluation using the MADRS scale and an ECT group—TRD patients (*n* = 10) who received 12 ECT treatments over four weeks (with a one-day intertreatment interval) following blood sampling and clinical evaluation at baseline and then once a week (Figure 2). The inclusion criteria are as follows: persons aged 18 to 75, both sexes, the existence of treatment-resistant MDD (i.e., inadequate response to a minimum of two antidepressants despite adequacy of the treatment trial and adherence to treatment [10]) in whom ECT was considered clinically appropriate, MADRS score above 20, and voluntary participation of the subjects in this study. The exclusion criteria are as follows: persons younger than 18 years and older than 75 years; patients who have received ECT in the past 6 months; refusal of subjects to participate in this study; pregnancy or breastfeeding; and the existence of any disease, condition, or circumstances that, in the judgment of the researcher, interfere with the subject’s participation in this study and adherence to study procedures. The subject could be excluded from the study at any time according to the decision of the subject itself, individual clinical judgment of the researcher, or the request of the competent regulatory bodies. The diagnosis of TRD and the indication and implementation of ECT treatment were performed by a certified specialist in psychiatry, according to a valid, well-founded methodological approach.

ECT was applied three times per week with a Thymatron® System IV device (Somatics, LLC, Lake Bluff, IL, USA) using bitemporal electrode placement, which was the standard approach at the study centre at the time of the study and is considered the most effective ECT regimen [14,23]. The study protocol did not affect ECT parameters such as ECT frequency, duration of ECT course, applied electrical stimulus, or electrode position. All participants started ECT treatment in inpatient conditions. Anaesthesia and muscle relaxation were achieved using propofol (1.0–1.5 mg/kg i.v.) and succinylcholine (0.5–1.25 mg/kg i.v.). The initial stimulus dose was set according to the appropriate guidelines and was increased during the ECT course depending on the ECT application response. None of the subjects experienced any adverse events that affected their participation in the study.

Evaluation of clinical response to therapy was performed using the Montgomery Aschberg Depression Rating Scale (MADRS), a clinician-rated scale designed to measure depression severity and detect changes during the treatment course [24]. The scale consists of 10 items, each of which is scored from 0 (symptom not present or normal) to 6 (severe or continuous presence of the symptom), for a total possible score of 60.

### 2.1. Blood Collection and PBMC Isolation

Ten milliliters of whole blood were collected from each patient and healthy control via venipuncture into a BD vacutainer EDTA tube (BD, 366643 Franklin Lakes, NJ, USA,). PBMC isolation was performed using Lymphocyte Separation Medium 1077 (PromoCell, c-44010, Heidelberg, Germany), according to the manufacturer’s instructions. Briefly, whole blood samples were diluted to 1:1 with a balanced salt solution, and this was then layered on top of the Lymphocyte Separation Medium in a 50 mL conical tube, followed by centrifugation at 440× *g* for 40 min at room temperature, to separate the buffy coat, which was transferred to a fresh tube. If the RBC lysis was required, the RBC lysis buffer (BioLegend, 420301, San Diego, CA, USA) was added for 5 min on ice. Following the washing, the isolated PBMCs were cryopreserved in 90% FBS/10% DMSO until RNA extraction.

### 2.2. RT-PCR Analysis

Collected samples were thawed briefly in a 37 °C water bath and rinsed with pure FBS. The RNA was isolated using the GeneMATRIX Universal RNA Purification Kit (EURx, E3598, Gdańsk, Poland) following the manufacturer’s instructions. The RNA concentration and purity were determined using the Eppendorf^®^ Biophotometer (Eppendorf, Hamburg, Germany).

For reverse transcription, iScript Reverse Transcription Mastermix (Bio-Rad, Hercules, CA, USA) was used. Real-time PCR was carried out using SsoAdvanced Universal SYBR Green Supermix (Bio-Rad, Hercules, CA, USA) and mRNA-specific primers (Supplement S1) for Bax, Bcl-2, p53, cytochrome c, and β-actin as a housekeeping gene (Invitrogen, Waltham, MA, USA). Quantitative RT-PCR reactions were performed using Bio-Rad CFX96 Real-Time PCR (Bio-Rad, Hercules, CA, USA), and after data analysis, the relative gene expression was calculated according to Livak and Schmittgen [25].

### 2.3. Statistical Analysis

The comparison between healthy subjects and patients was performed using Student’s *t* test or the Wilcoxon–Mann–Whitney U test, according to the type of data distribution. The comparisons between estimated parameters in predefined time-framed assessments in TRD patients were performed by using repeated measures ANOVA or the Friedman test, according to the type of data distribution. Between-group comparisons for two related samples in TRD patients were performed using the Bonferroni post hoc test or the Wilcoxon Signed Rank Test, according to the type of data distribution. Kendall’s Tau-b correlation was used to analyze the relationship between parameters obtained in blood samples and clinical outcome. The value of *p* < 0.05 was considered to be significant. Statistical analysis was performed with SPSS version 20.0 statistical package (IBM SPSS Statistics 20).

## 3. Results

**Figure 3:**
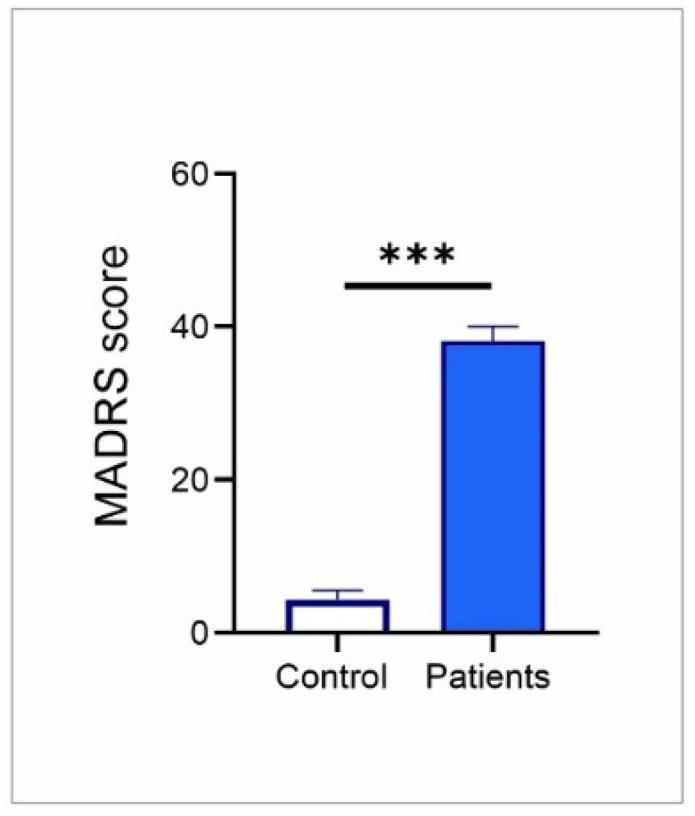
MADRS scores in healthy controls (n = 15) and baseline values for TRD patients (prior to ECT protocol, n = 10). Values are expressed as mean ± S.E.M., *** *p* < 0.001.

As shown in Figure 3, baseline (prior to ECT protocol) MADRS scores in TRD patients were markedly higher (almost tenfold) when compared to healthy controls (Mann–Whitney U test, Z = 4.2, *p* < 0.001), reflecting greater severity of depressive symptoms and confirming that this elevation in MADRS score is consistent with the clinical profile typically observed in depressive disorders.

**Figure 4:**
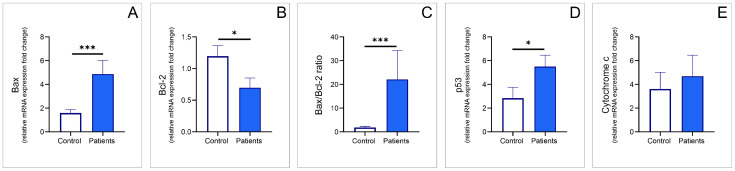
The relative gene expression fold change in healthy controls (n = 15) and baseline values for TRD patients (prior to ECT protocol, n = 10) (**A**) Bax; (**B**) Bcl-2; (**C**) Bax/Bcl-2 ratio; (**D**) p53; (**E**) cytochrome c. Values are expressed as mean ± S.E.M., * *p* < 0.05, *** *p* < 0.001.

The blood sample analyses for the apoptosis-related relative mRNA expression (Figure 4) also showed significant differences for some evaluated apoptotic markers. A threefold increase in Bax relative mRNA expression (Figure 4A) was observed in the group of TRD patients before the initiation of the ECT protocol, when compared to healthy volunteers (Mann–Whitney U test, Z = −3.8, *p* < 0.001), which was accompanied by a 50% reduction in Bcl-2 relative mRNA expression (Figure 4B, *t*-test, df = 23, *p* = 0.049). Such a response was also evident in their ratio (Figure 4C, Mann–Whitney U test, Z = −3.4, *p* < 0.001). Similarly to Bax, the relative mRNA expression of p53 in TRD patients was significantly increased compared to the control group (Figure 4D, Mann–Whitney U test, Z = −2.6, *p* = 0.011). As shown in Figure 4E, relative cytochrome c mRNA expression showed no significant difference between patients and healthy subjects.

**Figure 5:**
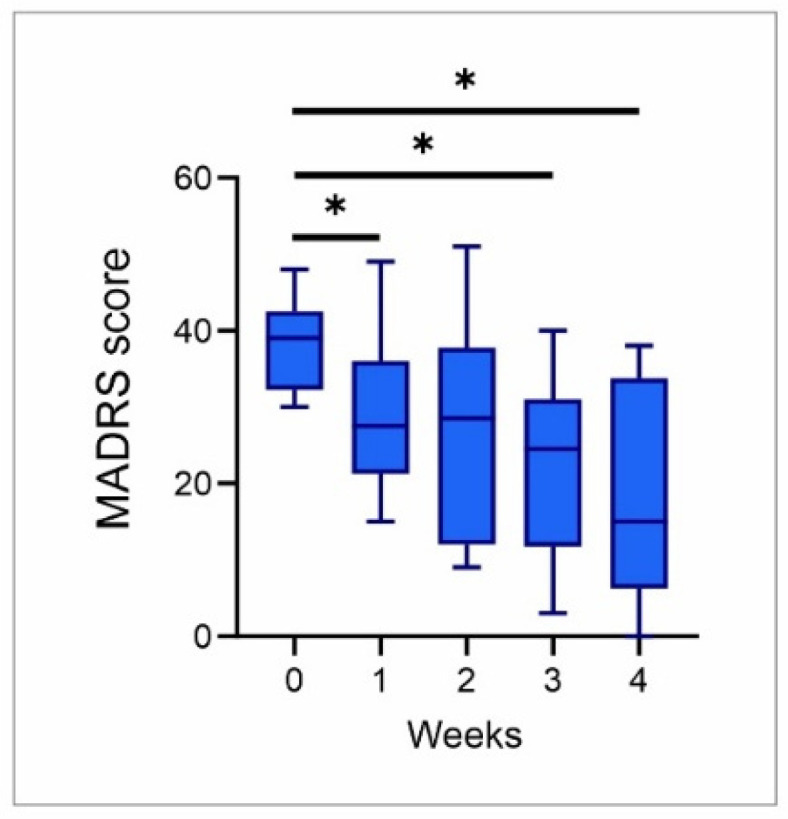
Changes in MADRS score during a 4-week ECT protocol in TRD patients. Values are expressed as median values for 10 patients, * *p* < 0.05.

Changes in the MADRS score of TRD patients during a 4-week ECT protocol, presented in Figure 5, following the algorithm manifested as an almost stepwise decrease [F(2.63, 23.67) = 8.3, *p* = 0.001]. Significant clinical improvement (*p* < 0.05) was observed even after one week of treatment, and a gradual decline was continued (except in week 2) until the end of the 4-week protocol (*p* < 0.05).

**Figure 6:**
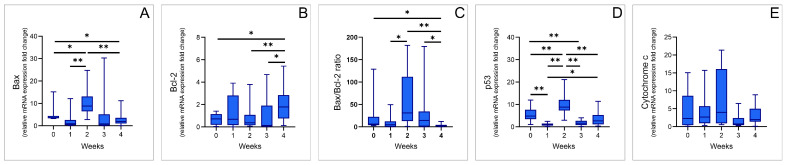
Changes in apoptotic markers relative gene expression fold change during the 4-week ECT protocol in TRD patients (**A**) Bax; (**B**) Bcl-2; (**C**) Bax/Bcl-2 ratio; (**D**) p53; and (**E**) cytochrome c. Values are expressed as median values for 10 patients, * *p* < 0.05, ** *p* < 0.01.

More complex responses to ECT were observed for apoptotic markers of relative mRNA expression (Figure 6). Bax relative mRNA expression (Figure 6A, χ2 = 16.0, df = 4, *p* = 0.003) declined only after one week of treatment, although not significantly (Z = −1.9, *p* = 0.059). However, the initial response was followed by a significant increase after two weeks of ECT protocol implementation, reaching values above the baseline (Z = −2.0, *p* = 0.047). The continuation of ECT trials resulted in the subsequent decline in Bax mRNA expression that, after three weeks, was significantly lowered compared to week 2 and, finally, resulted in significantly lower values compared to initial after completing the four-week protocol (Z = −2.5, *p* = 0.013). Much simpler alterations in Bcl-2 relative mRNA expression (Figure 6B, χ2 = 9.6, df = 4, *p* = 0.048) were recorded during the applied ECT protocol. Namely, there was a significant increase in Bcl-2 mRNA expression, starting after two weeks of protocol, reaching the highest values at the end of the ECT protocol (Z = −2.5, *p* = 0.013). The relationship between Bax and Bcl-2 relative mRNA expression, as shown in Figure 6C (χ2 = 16.0, df = 4, *p* = 0.003), was probably due to the “rebound” phenomenon expressed in Bax relative mRNA expression, showing a final significant decline (Z = −2.2, *p* = 0.028), which was not manifested by the clear trendline after week 2 and 3 of the employed ECT trial. Again, like alterations for Bax, p53 relative mRNA expression (Figure 6D: χ2 = 30.2, df = 4, *p* < 0.001) also resulted in a significant decline after three weeks of ECT trial when compared to the baseline (Z = −2.8, *p* = 0.005), but with a transient increase after two weeks of protocol. In contrast, the applied ECT protocol did not significantly affect cytochrome c relative mRNA expression (Figure 6E).

**Figure 7:**
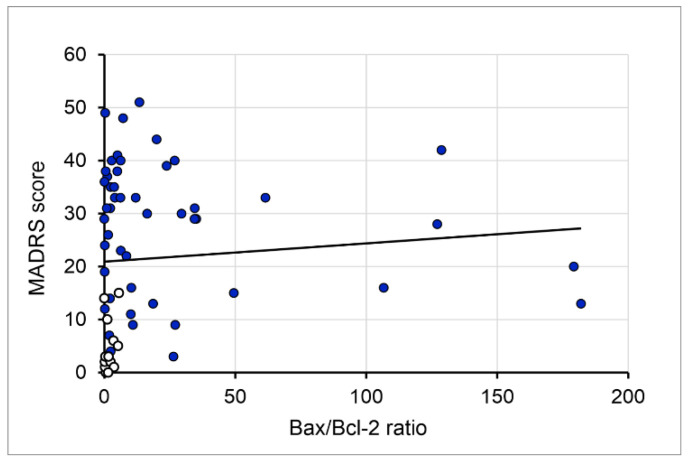
The relationship between the Bax/Bcl-2 ratio and MADRS score in healthy controls (white dots) and TRD patients (blue dots).

Finally, by choosing the Bax/Bcl-2 ratio (a marker of the quantitative relationship between pro- and anti-apoptotic indicators) and MADRS scores (Figure 7), we estimated the relationship between evaluated markers in peripheral blood samples and clinical manifestations. Simple regression analysis revealed that the Bax/Bcl-2 ratio was positively correlated with MADRS scores (rτ = 0.174, *p* = 0.042).

## 4. Discussion

Apoptosis-related pathways may play a key role in MDD pathophysiology and treatment response, making the study of ECT’s impact on related gene expression crucial for understanding its therapeutic effects.

The results of our study, measuring clinical response using MADRS, showed significant improvement, i.e., more than 50% reduction of baseline values, by the end of the ECT protocol (according to predefined criteria [26]). The obtained clinical results are in line with previous trials, confirming that ECT seems to be an effective treatment for severely depressed patients, as well as for patients with previous pharmacotherapy failure [27,28,29,30]. Interestingly, unlike the parameters obtained from blood samples, the scoring of clinical outcome showed a continual and gradual decrease in each subsequent week of the ECT protocol.

Our results demonstrate a dynamic, treatment-phase-dependent regulation of mitochondrial apoptotic signaling during ECT that correlates with clinical improvement (as measured by the MADRS) in TRD. At baseline, TRD patients exhibited enhanced pro-apoptotic mRNA expression, specifically showing increased levels of Bax, p53, and cytochrome c, whereas the mRNA of the anti-apoptotic Bcl-2 was decreased compared to healthy controls. Our findings are consistent with prior research indicating a significant rise in Bax and a reduction in Bcl-2 mRNA expression levels within PBMCs of patients with MDD, especially those at high risk for suicide, relative to healthy controls [5].

Also, our results demonstrated that during ECT, the mRNA expression of the Bax/Bcl-2 ratio and p53 followed a phased trajectory: an early decrease in the first week, a transient rebound increase in the second week, and then a decline again in the third and fourth weeks. The mRNA expression of cytochrome c exhibited a similar phase trajectory; however, the observed changes were not statistically significant. The decrease in the Bax/Bcl-2 ratio also correlated with an improvement in MADRS. Our results align with the dynamic, stage-dependent biological responses previously described during ECT treatment, indicating complex neurochemical, neurobiological, and neuroendocrine changes in patients undergoing ECT [31,32]. The early decrease in pro-apoptotic markers’ mRNA expression following the first week of ECT sessions may represent an immediate adaptive response to the physiological stress induced by ECT. It has been established that ECT triggers significant neuroendocrine activation, including a transient elevation in cortisol levels [18,33] and sympathetic stimulation (ACTH, EPI, NA, and prolactin) [34,35,36], which regulate the HPA axis and the acute pro-inflammatory response, particularly the increases in IL-6 [37,38]. All of these are known to temporarily suppress pro-apoptotic gene transcription in peripheral immune cells (such as neutrophils and T lymphocytes) to prolong their survival and enhance the immediate immune response, as part of a rapid decrease in the Bax/Bcl-2 mRNA expression ratio and a cell-protective response. Many studies have well-established that the acute and short-term stress release of catecholamines and cortisol increases the total blood cell number and induces a rapid and significant redistribution of immune cells (stress-induced leukocytes) among different body parts, which may be an essential survival response [39]. Additionally, high acute cortisol might induce the sequestration of lymphocytes into secondary lymphoid organs, thereby suppressing apoptosis and inflammation within injured areas of the brain [40]. Furthermore, many researchers demonstrated that mature single positive CD4+ or CD8+ cells are protected from cortisol (glucocorticoids)-induced apoptosis by CD28 co-stimulation or increased Bcl-2 and Bcl-xL protein expression, thereby suppressing Bax activation [41,42]. Remarkably, a single stimulation with ECT can suppress apoptosis, as well as induce proliferation, within the dentate gyrus neuron layer, which can survive for months [43]. Functionally, our findings suggest that a decreased Bax/Bcl-2 mRNA expression ratio lowers susceptibility to mitochondrial apoptosis and cytochrome c release following ECT stimulation, and it may help inhibit apoptosis after the initial ECT insult. Hence, previous research had revealed that the level of expression of Bcl-2 proteins changed quickly in relation to acute stress, with a brief increase in anti-apoptotic Bcl-2, suppressing Bax activation and p53 activation [44].

Following ECT stimulations during the second week, there was a significant increase in the mRNA Bax/Bcl-2 expression ratio and p53 levels; however, there was a statistically nonsignificant increase in cytochrome c mRNA expression. These results may suggest the activation of mitochondrial remodeling and the initiation of cellular adaptation mechanisms following second-week ECT sessions, rather than the actual onset of apoptosis. This regulated increase in pro-apoptotic BAX and p53 gene expression could promote specific MOMP/mitochondrial autophagy, leading to the removal of defective cellular components instead of cell death [45].

ECS has been shown to effectively treat severe depression by inducing seizures that strongly induce autophagy, primarily via pathways such as AMPK, which can be accompanied by transient Bax activation and help in the clearance of cellular debris, promotion of neuronal survival, and stimulation of neuroplasticity [46]. The treatment of ECT in TRD patients has been evidenced by many studies to play a significant role in the regulation of mitochondrial dynamics and in the improvement of mitochondrial function, which encompasses not only respiratory activity but also intracellular mitochondrial load in both neurons and PBMCs [47]. Moreover, recurring seizures can trigger a cellular stress response that activates the tumor suppressor protein p53, which subsequently increases pro-apoptotic genes such as Bax, Puma, Noxa, and Bid while reducing Bcl-2, which could trigger apoptosis (Figure 1). Conversely, p53 is also involved in the regulation of DNA repair, metabolic alterations, mitochondrial function, and autophagy, thus signaling either the survival or the removal of damaged neurons. This highlights the complex role of p53 in how neurons respond to stress triggered by seizures [48].

The basic concept of ECT is to produce a generalized grand mal seizure (approximately 30 s). Seizures that are prolonged, such as status epilepticus (cerebral seizures for more than 30 min), are recognized to trigger neuronal apoptosis or necrosis, especially in the hippocampus, by means of excitotoxicity, a calcium overload induced by glutamate release [49]. It is therefore reasonable to assume that some neuronal death may occur during short-duration ECT seizures. The different studies showed that ECS can activate microglial cells, astrocytes, neuro-inflammations, cytokines, oxidative responses, and apoptotic neuronal cells to regulate the adaptive immunity mechanisms that protect against chronic neuronal damage [50,51]. Therefore, inflammatory mediators released by glia represent a rapid way to eliminate damaged neurons by activating M2-type microglia, which leads to long-term neuroprotection [52]. However, these effects are rapid and may be temporary, with repeated ECT treatments normalizing them, indicating the activation of neuroprotection. Some studies suggest that rapid increases in reactive microglia and inflammatory pathways collaborate to prevent oxidative stress induced-neuronal apoptosis to rescue and support injured neurons in early brain damage [53]. Interestingly, an earlier study found that ECS stimulus induces small (10%) but statistically significant neuronal death in the field CA1 and the dentate gyrus seven days afterwards; however, caspase 3 activity was significantly decreased in the hippocampus, suggesting that most cell death in field CA1 occurred rapidly after the first ECS sessions [19]. Since neurogenesis has been shown to increase in the hippocampal dentate gyrus (DG) of depressed patients who received ECT treatment, the authors also suggest that cell death in a minority of cells may be the stimulus for increased neurogenesis as a compensatory mechanism in a variety of lesions [54]. Moreover, our biphasic changes in pro-apoptotic markers in ECT-treated TRD patients correspond with the previous study, indicating that a single ECS enhanced hippocampal cell proliferation and anti-apoptotic effects. On the contrary, the number of apoptotic cells increased after 10 ECS treatments when compared with both single and 20 ECS treatments [20]. Hence, the increase in the mRNA levels of Bax and p53 observed in the second week of ECT treatments in our study might indicate a temporary rebound, which might be dominated by controlled, non-pathological mitochondrial quality control. This mechanism removes injured neurons and peripheral blood cells, as well as defective mitochondria that could cause mitochondrial-dependent apoptosis after ECT treatments [55]. Therefore, our results support previous data that indicated adaptive mitochondrial biogenesis may occur after the second week of ECT treatments.

In weeks three and four of ECT treatments, mRNA levels of Bax and p53 decreased while Bcl-2 increased, indicating cellular homeostasis normalization and clinical stabilization. As repeated ECT treatments in TRD reduce depressive symptom severity and regulate systemic stress, mitochondrial and apoptotic signaling tend to progress toward an anti-apoptotic condition [56]. Earlier studies indicated that effective ECT leads to Bcl-2 family balance shifted toward an antiapoptotic effect, thus making the cells less prone to apoptosis and more resistant to damage. Specifically, the anti-apoptotic effect of ECS was specifically evidenced by data indicating that, when repeated unpredictable stress (RUS) was applied repeatedly, the expression of Bcl-2 and Bcl-xL mRNA decreased, whereas the prolonged ECS treatment resulted in an increased expression of Bcl-2 mRNA in the hippocampus, CeA, Cg, and Fr, as well as of Bcl-xL mRNA in the hippocampus [57]. Additionally, another study indicated that repeated ECS prevented status epilepticus (SE) neuronal cell death in rats by reducing apoptosis-like neuronal morphology, DNA fragmentation, and pro-apoptotic Bcl-XS protein and mRNA in the hippocampus and rhinal cortex [58]. Furthermore, ECS may prevent apoptosis by activating survival signal pathways and inhibiting pro-apoptotic signaling. In animals, recurrent ECS increases proliferative signals, including the Cdk2-pRB-E2F1 cell cycle pathway [59]. An earlier study has demonstrated that the Cdk2-pRB-E2F1 and p53 pathways function synergistically as the key regulators of neuronal survival or death [60]. Therefore, our results corroborate previous findings that repeated ECT significantly reduced p53 mRNA levels, preventing apoptosis and enhancing cell survival. Another study reported that after 10 days, ECS lowered proapoptotic c-Myc expression and BAD activation, which could lead to activation of anti-apoptotic Bcl-2 in rats’ frontal cortex, thus reducing neuronal apoptosis [61]. C-Myc overexpression has been shown to induce apoptosis by enhancing the mRNA levels of the endogenous pro-apoptotic Bax [62,63], alongside the apoptosis in the mitochondria caused by Bcl-2/BclXl suppression, BAX and p53 protein increase, and ultimately, cytochrome c release [64,65,66]. Thus, our results confirm previous findings that long-term ECT treatment decreased mitochondrial apoptosis via inhibiting p53 and downregulating Bax and upregulating Bcl-2 mRNA levels.

Although this study convincingly demonstrates peripheral changes in apoptosis-related gene expression, a more accurate scientific approach would benefit from a broader systems-level framework linking mitochondrial apoptosis, neuroinflammation, stress physiology, and brain homeostasis. It is worth noting that ECT is increasingly highlighted as a global neuromodulatory intervention affecting sleep architecture, metabolic clearance, inflammatory load, and network-level plasticity [67,68]. Even more, it has been reported that ECT protocols result in modulation of sleep, neuroendocrine stress responses, and inflammatory signaling that may secondarily enhance glymphatic clearance (reducing mitochondrial stress and apoptotic signaling over time [67,68]). It seems that impaired glymphatic function is closely related to sleep architecture, neuroinflammation, mitochondrial stress, and metabolic waste clearance, which may represent a convergent pathophysiological pathway in mood disorders and is also affected by specific therapeutic protocols.

Mostly for practical reasons, peripheral biomarkers are widely used in the estimation of numerous neurologic [69] and psychiatric disorders [70], including MDD [71]. Although it is obvious that the values of those biomarkers from peripheral blood can be affected by numerous factors and may not necessarily represent the pathophysiological processes occurring in the specific brain regions, their accessibility not only favors them to be widely used in clinical practice but also in research protocols.

In summary, the triphasic pattern of Bax and p53 mRNA expression observed in our study suggests an interplay involving initial suppression of cellular stress during ECT, followed by adaptive cellular and mitochondrial remodeling that occurs after the second week of ECT, ultimately leading to the normalization of cellular homeostasis by the end of the ECT treatments. The observed gene expression pattern of apoptotic markers corresponds to the clinical outcomes and reinforces the theory that ECT might modulate the mitochondrial apoptotic signaling pathway, thus leading to the suppression of apoptosis. Indeed, the observed algorithm manifested as biphasic and triphasic molecular responses during ECT may be the groundwork for the conceptual synthesis. We find that additional investigations (which will include a larger sample size, additional parameters, and responses to prolonged ECT protocols) should be employed prior to drawing explicit conclusions.

### Limitations of This Study

As a pilot study, the sample size was small and represented a heterogeneous group of patients in terms of age, MDD characteristics, and previous therapeutic protocols. Also, the investigation is based on a limited number of methods, and findings should be interpreted with caution. The absence of long-term follow-up limits conclusions about the effects of the applied therapeutic protocol. Despite these limitations, the observed changes offer a foundation for future investigations and support the feasibility of targeting specific neurochemical alterations accompanied by the clinical outcome of ECT.

## 5. Conclusions

The biological mechanisms through which ECT exerts therapeutic effects in MDD remain insufficiently understood. Although this investigation offered convincing evidence for the impact of apoptosis-related indicators in response to ECT, it seems necessary to analyze the other pathophysiological pathways associated with MDD (mitochondrial dysfunction, oxidative damage, inflammation, etc.), and their interplay with apoptotic mechanisms.

Future investigations, which will include a sufficient number of patients, may be considered essential for establishing the neurochemical alterations algorithm that may be used for a personalized approach, including the potential adaptations of ECT trials and/or adequate pharmacological pretreatment.

## Data Availability

The data will be available upon reasonable request.

## Supplementary Materials

The following supporting information can be downloaded at: https://www.mdpi.com/article/10.3390/genes17010057/s1, File S1. List of primers used for RT-PCR.

## Abbreviations

The following abbreviations are used in this manuscript:

ECT	Electroconvulsive therapy;
TRD	Treatment-resistant depression;
MADRS	Montgomery–Åsberg Depression Rating Scale;
MDD	Major depressive disorder;
ECS	Electroconvulsive stimulation;
E2F1	Early region 2 promoter transcription factor 1;
EDTA	Ethylenediaminetetraacetic acid;
PBMCs	Peripheral blood mononuclear cells;
FSB	3,3′-[(2-fluoro-1,4-phenylene)di-(1E)-2,1-ethenediyl]bis-6-hydroxy-benzoic acid;
DMSO	Dimethyl sulfoxide;
RT-PCR	Reverse transcription polymerase chain reaction;
MOMP	Mitochondrial outer membrane permeabilization;
AMPK	Adenosine monophosphate-activated protein kinase;
RUS	Repeated unpredictable stress;
Cdk2-pRB-E2F1	Cyclin-dependent kinase 2–retinoblastoma protein–E2F1 pathway.
