# Supplementary material for: The Impact of Electroconvulsive Therapy on Apoptosis-Related Biomarker Gene Expression in Treatment-Resistant Depression
Source: Genes (Basel). 2026 Jan 4;17(1):57. doi: 10.3390/genes17010057 (PMC12840669; doi:10.3390/genes17010057)
Supplement: Supplementary file 1 [file genes-17-00057-s001.zip › genes-4080452-supplementary.pdf]

List of primers used for RT-PCR

| **Primer Name** | **Sequence (5'- 3')** | **Product size (bp)** | **Tm (°C)** |
|---|---|---|---|
| Human β-actin | F CTCACCATGGATGATGATATCGC<br>R AGGAATCCTTCTGACCCATGC | 163 | 59 |
| Human Bax | F ATGGACGGGTCCGGGGAGCA<br>R CCCAGTTGAAGTTGCCGTCA | 175 | 64 |
| Human Bcl-2 | F CTTTGAGTTCGGTGGGGTCA<br>R GGGCCGTACAGTTCCACAAA | 162 | 60 |
| Human p53 | F TGCGTGTGGAGTATTTGGATG<br>R TGGTACAGTCAGAGCCAAGGTG | 181 | 60 |
| Human Cytochrome c | F GGGCGAGAGCTATGTAATGCAAG<br>R TACAGCCAAAGCAGCAGCTCA | 132 | 62 |

F - *forward*; R – *reverse.*
